# Systematic review of latent tuberculosis infection research to inform programmatic management in Ireland

**DOI:** 10.1007/s11845-021-02779-w

**Published:** 2021-09-30

**Authors:** James O’Connell, Eoghan de Barra, Samuel McConkey

**Affiliations:** 1 School of Postgraduate Studies, Royal College of Surgeons in Ireland, Dublin 2, Dublin, Ireland; 2 Department of International Health and Tropical Medicine, Royal College of Surgeons in Ireland, Dublin 2, Dublin, Ireland; 3 Department of Infectious Diseases Medicine, Beaumont University Hospital, Dublin 5, Dublin, Ireland

**Keywords:** Health protection, Latent tuberculosis, Mycobacterium tuberculosis, Republic of Ireland, Screening

## Abstract

The World Health Organisation (WHO) End Tuberculosis (TB) Strategy and the WHO Framework Towards Tuberculosis Elimination in Low Incidence Countries state that latent tuberculosis infection (LTBI) screening and treatment in selected high-risk groups is a priority action to eliminate TB. The European Centre for Disease Prevention and Control (ECDC) advises that this should be done through high-quality programmatic management, which they describe as having six key components. The research aim was to systematically review the literature to identify what is known about the epidemiology of LTBI and the uptake and completion of LTBI screening and treatment in Ireland to inform the programmatic management of LTBI nationally. A systematic literature review was performed according to a review protocol and reported in adherence with the Preferred Reporting Items for Systematic Reviews and Meta-Analysis (PRISMA) statement. Twenty-eight studies were eligible for inclusion and described LTBI screening or treatment performed in one of five contexts, pre-biologic or other immunosuppression screening, people living with HIV, TB case contacts, other vulnerable populations, or healthcare workers. The risk of bias across studies with regard to prevalence of LTBI was generally high. One study reported a complete cascade of LTBI care from screening initiation to treatment completion. This systematic review has described what published research there is on the epidemiology and cascade of LTBI care in Ireland and identified knowledge gaps. A strategy for addressing these knowledge gaps has been proposed.

## Introduction

Reactivation of latent tuberculosis (TB) infection (LTBI) is a significant challenge for global TB elimination efforts. It is estimated that 23% of the world’s population and 13.7% of Europe’s population have LTBI [1]. The World Health Organisation’s (WHO) End TB Strategy and Framework Towards Tuberculosis Elimination in Low-Incidence Countries state that LTBI screening and treatment in selected high-risk groups are priority actions to eliminate TB [2, 3]. The European Centre for Disease Prevention and Control (ECDC) advises that this should be done through high-quality programmatic management, which they describe as having six key components (Table 1) [4].

**Table 1:** Key components for the programmatic management of latent tuberculosis infection

	Component
1	Identification of groups at risk of having LTBI or an increased risk of progressing to active TB. These target groups should be prioritized for LTBI screening and treatment
2	Definition of diagnostic approach for LTBI detection, including both the selection of diagnostic test(s) and the diagnostic algorithm most appropriate for each target group
3	Provision of LTBI treatment using treatment regimens that are effective and promote adherence and completion by different target groups
4	Implementation of patient-centred strategies for service delivery
5	Effective health education and communication with target groups and health care providers
6	Programme monitoring and evaluation

*LTBI* latent tuberculosis infection

Risk groups with a high prevalence of LTBI or a high risk of TB reactivation should be prioritised for LTBI screening and treatment [2–4]. For some cohorts, whether programmatic LTBI screening and treatment occurs depends on the country-specific epidemiology of LTBI and the resources available for screening and treatment (Table 2) [3, 4]. As well as identifying cohorts who should be screened and treated for LTBI, it is important to know whether programmatic LTBI management in these cohorts is feasible by having prior knowledge of the uptake and completion of LTBI screening and treatment (known as the cascade of care) and having considered its cost and cost-effectiveness [4].

**Table 2:** Programmatic screening and treatment of latent tuberculosis infection in countries with a low incidence of TB [3, 4]

Cohorts which should be programmatically screened and treated in all EU/EEA countries	Cohorts where programmatic screening and treatment is dependent on the country specific LTBI epidemiology and resources available
Immunosuppressed persons (such as patients on anti-TNF alpha treatment),	People who are homeless
People living with HIV (regardless of CD4 cell count or HIV antiretroviral therapy status)	People who use drugs
Patients preparing for transplantation	Prison inmates
Patients with end-stage renal diseases and/or preparing for dialysis	Immigrants from countries with a high TB incidence
Patients with silicosis; people with pulmonary fibrotic lesions	Health care workers
Contacts of infectious TB cases (based on a risk assessment of their exposure)	

*EU* European Union, *EEA* European Economic Area, *TNF* tumour necrosis factor, Area: *CD4* cluster of differentiation 4, *LTBI* latent tuberculosis infection

Many countries with a low incidence of TB are establishing programmatic LTBI management to achieve TB elimination after researching the prevalence of LTBI in different cohorts and the feasibility of programmatic LTBI management. In the United Kingdom (UK), they have identified that immigrants from countries with a very high incidence of TB contribute significantly to the case burden nationally [5, 6]. They have demonstrated a high prevalence of LTBI among these immigrant cohorts and demonstrated that the rate of TB reactivation over time was significant, suggesting that TB reactivation, as opposed to primary TB infection, explained the high TB incidence in this cohort [7, 8]. Furthermore, they have researched the feasibility, acceptability and cost effectiveness of different screening strategies among high-risk immigrant cohorts [9–12]. Public Health England has established a national LTBI testing and treatment program for immigrants from countries with a high incidence of TB informed by their research on the prevalence of LTBI and feasibility of programmatic screening in this cohort [13]. This was a key action of their national collaborative strategy for TB [14]. Evidently, LTBI epidemiological and cascade of care research informed and enabled Public Health England to establish programmatic LTBI management in a target risk cohort.

The aim of this systematic review was to identify what is known about the epidemiology and cascade of care of LTBI in Ireland to inform its programmatic management nationally.

## Methods

A systematic literature review was performed according to a review protocol and reported in adherence with the Preferred Reporting Items for Systematic Reviews and Meta-Analysis (PRISMA) statement (Appendix 1) [15]. The protocol for this systematic review was registered with the Open Science Framework (https://doi.org/10.17605/OSF.IO/8ED29) and is available in Appendix 2.

Studies eligible for inclusion were those that described any group of patients who were screened or treated for LTBI in Ireland and reported using any one or a combination of chest radiography, tuberculin skin resting (TST) or interferon-gamma release assay (IGRA) testing to screen for LTBI. Studies had to report at least one of the following outcomes (chosen because they describe the cascade of LTBI care): the proportion of people screened out of the target population, the prevalence of a positive screening test in the target population, the proportion of those diagnosed with LTBI who were offered treatment, the proportion of those diagnosed with LTBI who started treatment for LTBI, the proportion of those diagnosed with LTBI who completed treatment out and the cost of performing screening or treatment of LTBI cases identified.

Clinical audits, randomized controlled trials, diagnostic accuracy studies, retrospective cohort reviews and prospective cohort reviews published between the 1st of January 2000 and the 31st of December 2019 (inclusive) were eligible for inclusion. Studies published in languages other than English were not eligible for inclusion. Studies where it was not possible to extract data on patients screened in Ireland alone were excluded.

A search of MEDLINE (via OVID), Embase, Web of Science, Google Scholar and published abstracts from national conferences in Ireland was conducted (search strategy is described in Appendix 2, date of last search: 14th of May 2020). The references of included studies were also searched. The literature search and data extraction were each conducted independently by two reviewers, and any disagreements relating to study eligibility or data extraction were resolved by discussion and mutual agreement. For the prevalence of a positive screening test, the risk of bias was assessed using a tool designed for TB prevalence studies that was derived from on an existing tool for prevalence studies (Appendix 3) [16].

## Results

### Search results

**Fig. 1:**
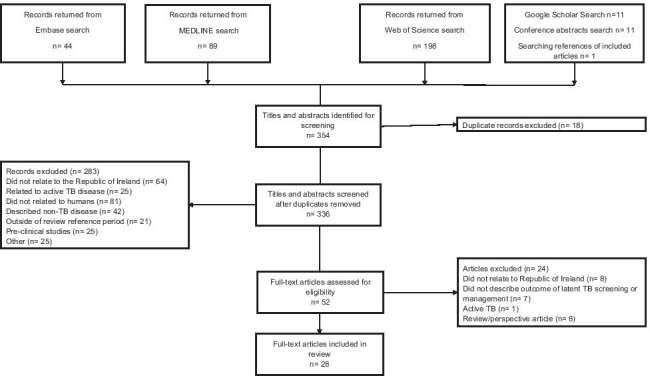
Flow diagram of literature search

The results of the search are described in Fig. 1. Fifty-two articles were identified for full-text review from the search of the indexed literature, Google Scholar, conference abstract searches, and the references of included articles. In total, 28 studies were identified as meeting the review inclusion criteria.

### Characteristics of included studies

The included studies described LTBI screening or treatment performed in one of five contexts (Table 3), pre-biologic or other immunosuppressive treatment screening (11 studies [17–27]), people living with human immunodeficiency virus (PLWHIV) (two studies [28, 29]), TB case contacts or prior to Bacillus Calmette–Guérin (BCG) vaccination (nine studies [30–38]), other vulnerable populations (two studies in asylum seekers [39, 40]) or health care workers (five studies [29, 41–44]). Most studies (19/28) were retrospective cohort reviews, seven were prospective cohort reviews and for one study, the design was unclear. All studies were performed on a regional or local level. Fourteen of 28 studies were conducted in tertiary care centres, nine were conducted in public health departments, and three were in secondary care centres.

**Table 3:** Characteristics of studies included

Study	Study design*	Setting	Cohort (cohort size)
Gnanasekaran et al. [17]	Retrospective	Secondary centre	Pre-biologic screening in patients with IA (*n* = 39)
O’Flynn et al. [18]	Retrospective	Tertiary centre	Pre-biologic screening in patients with IA (*n* = 70)
Awan et al. [19]	Prospective	Secondary centre	Pre-biologic screening in patients with IA (*n* = 25)
O’Flynn et al. [20]	Prospective	Tertiary centre	Pre-biologic screening in patients with IA (*n* = 109)
Hurley et al. [21]	Retrospective	Tertiary centre	Pre-organ transplantation screening (*n* = 101)
Safwat et al. [22]	Retrospective	Secondary centre	Pre-biologic screening (43% of cohort) (*n* = 78)
Haroon et al. [23]	Prospective	Tertiary centre	Pre-biologic screening in patients with IA (*n* = 132)
O’Flynn [24]	Unclear	Tertiary centre	Pre-biologic screening in patients with IA (*n* = 188)
Martin et al. [25]	Prospective	Tertiary centre	Pre-biologic screening in patients with IA (*n* = 150)
Jordan et al. [26]	Retrospective	Tertiary centre	Pre-biologic screening in patients with IA (*n* = 63)
Kelly et al. [27]	Retrospective	Tertiary centre	Pre-biologic screening in patients with psoriasis (*n* = 101)
Ní Cheallaigh et al. [28]	Prospective	Tertiary centre	People living with HIV (*n* = 256)
Ali et al. [29]	Retrospective	Tertiary centre	PLWHIV (*n* = 331), Occupational screening of new entrant health care workers (*n* = 2410)
Higgins et al. [30]	Retrospective	PHD	TB outbreak (6 cases) in the community (*n* = 268)
Glynn et al. [31]	Prospective	PHD	Contact tracing of 39 sporadic TB cases (*n* = 701)
O’Donovan et al. [32]	Prospective	PHD	TB outbreak (6 cases) in university students (*n* = 71)
O’Meara et al. [33]	Retrospective	PHD	TB outbreak (3 cases) in a primary school (*n* = 307)
O’Sullivan et al. [34]	Retrospective	PHD	TB outbreak (13 cases) in a secondary school (*n* = 1200)
Bambury et al. [35]	Retrospective	PHD	Contact tracing of 118 sporadic TB cases (*n* = 1082)
Gaensbaeur et al. [36]	Prospective	PHD	TB outbreak in two creches (*n* = 268)
Hennessy [37]	Retrospective	PHD	Children tuberculin skin tested before BCG (1854)
Tam et al. [38]	Retrospective	Tertiary centre	TB-related referrals to a specialist paediatric clinic (*n* = 13)
Millar et al. [39]	Retrospective	PHD	Asylum seekers attending communicable disease screening in Cork and Kerry (*n* = 4780)
Doyle et al. [40]	Retrospective	PHD	Asylum seekers undergoing communicable disease screening 1998–2003 (*n* = 236)
Smyth et al. [41]	Retrospective	Unknown	Health care workers with significant exposure to infectious TB (*n* = 41)
Kelly et al. [42]	Retrospective	Tertiary centre	Occupational screening of overseas health care workers (*n* = 505)
Power et al. [43]	Retrospective	Tertiary centre	Overseas nursing recruits from India (*n* = 54)
Arya et al. [44]	Retrospective	Tertiary centre	Health care workers with a positive TST referred to the TB clinic (*n* = 243)

*IA* inflammatory arthritis, *PHD* Public Health Department, *PLWHIV* people living with HIV

*All studies were cohort reviews

The reporting of the cascade of care was generally very incomplete (Table 4). Only six studies described the proportion of the target population that completed screening. Provider recommendation, patient acceptance and patient completion of LTBI treatment were reported in 10, 12 and eight studies, respectively. One of 28 studies reported the complete cascade of care from screening initiation to treatment completion [17]. One study reported an estimate of the cost of LTBI screening and treatment [26].

**Table 4:** Screening tests and outcomes reported in included literature

Study	Screening test(s) reported	Outcome(s) reported					
		Proportion screened	Proportion screened positive	Proportion offered prophylaxis	Proportion accepting prophylaxis	Proportion completing prophylaxis	Cost of screening/treatment
**Studies evaluating latent TB infection screening in immunosuppressed patients**							
Gnanasekaran et al. [17]	IGRA, CXR	Yes	Yes	Yes	Yes	Yes	No
O’Flynn et al. [18]	Unclear	No	Yes	No	No	No	No
Awan et al. [19]	IGRA, TST	No	Yes	Yes	Yes	Yes	No
O’Flynn et al. [20]	IGRA	No	Yes	Yes	Yes	No	No
Hurley et al. [21]	IGRA, TST	No	Yes	No	No	No	No
Safwat et al. [22]	IGRA, TST, CXR	No	Yes	No	No	No	No
Haroon et al. [23]	TST, CXR	No	Yes	Yes	Yes	Yes	No
O’Flynn 2012 [24]	IGRA, TST, CXR	No	Yes	Yes	Yes	No	No
Martin et al. [25]	IGRA, CXR	No	Yes	No	No	No	No
Jordan et al. [26]	IGRA, TST, CXR	No	Yes	Yes	Yes	Yes	Yes
Kelly et al. [27]	IGRA, TST, CXR	No	Yes	Yes	Yes	No	No
Proportion of studies reporting outcome		1/11	11/11	7/11	7/11	4/11	1/11
**Studies evaluating latent TB infection screening in people living with HIV**							
Ní Cheallaigh et al. [28]	IGRA, TST	No	Yes	Yes	No	No	No
Ali et al. [29]	TST, CXR	No	Yes	No	No	No	No
Proportion of studies reporting outcome		0/2	2/2	1/2	0/2	0/2	0/2
**Studies evaluating latent TB infection screening in recent TB contacts or prior to BCG vaccination**							
Higgins et al. [30]	TST	Yes	Yes	No	Yes	No	No
Glynn et al. [31]	Unclear	Yes	Yes	No	Yes	Yes	No
O’Donovan et al. [32]	Unclear	No	Yes	No	No	No	No
O’Meara et al. [33]	TST, CXR	Yes	Yes	No	No	No	No
O’Sullivan et al. [34]	TST, CXR	No	Yes	No	No	No	No
Bambury et al. [35]	Unclear	Yes	Yes	No	No	No	No
Gaensbaeur et al. [36]	TST, CXR	Yes	Yes	No	No	No	No
Hennessy [37]	TST, CXR	No	Yes	No	No	No	No
Tam et al. [38]	TST, CXR	No	No	Yes	No	Yes	No
Proportion of studies reporting outcome		5/9	8/9	1/9	2/9	2/9	0/9
**Studies evaluating latent TB infection screening in asylum seekers**							
Millar et al. [39]	Unclear	No	Yes	No	No	No	No
Doyle et al. [40]	TST, CXR	No	Yes	No	Yes	No	No
Proportion of studies reporting outcome		0/2	2/2	0/2	½	0/2	0/2
**Studies evaluating latent TB infection screening in health care workers**							
Ali et al. [29]	TST, CXR	No	Yes	No	No	No	No
Smyth et al. [41]	TST	No	Yes	No	No	No	No
Kelly et al. [42]	IGRA, TST	No	Yes	Yes	Yes	Yes	No
Power et al. [43]	TST, CXR	No	Yes	No	No	No	No
Arya et al. [44]	TST, CXR	No	No	No	Yes	Yes	No
Proportion of studies reporting outcome		0/5	4/5	1/5	2/5	2/5	0/5
Proportion of all studies reporting outcome		6/28	26/28	10/28	12/28	8/28	1/28

*TST* tuberculin skin test, *IGRA* interferon gamma release assay, *CXR* chest radiography

### Risk of bias assessment

The overall risk of bias in assessing the prevalence of LTBI in the included studies was high (Table 5, Appendix 3). Convenience sampling occurred in 25 of 26 studies. In 12 of 26 studies, there was a lack of a description of the patient exclusion criteria or how TB disease was identified. In 19 of 26 studies, the response rate, or the proportion of the target population who were screened, was not reported. Overall, the risk of bias was low (score 6–8) in two studies, moderate (score 3–5) in seven studies and high (score 0–2) in 17 studies.

**Table 5:** Risk of bias assessment

Study	Total risk of bias score	Risk of bias
**Studies evaluating latent TB infection screening in patients undergoing immunosuppression**		
Gnanasekaran et al. [17]	7	Low
O’Flynn et al. [18]	0	High
Awan et al. [19]	0	High
O’Flynn et al. [20]	2	High
Hurley et al. [21]	1	High
Safwat et al. [22]	1	High
Haroon et al. [23]	4	Moderate
O’Flynn [45]	4	Moderate
Martin et al. [25]	4	Moderate
Jordan et al. [26]	0	High
Kelly et al. [27]	4	Moderate
**Studies evaluating latent TB infection screening in people living with HIV**		
Ni Cheallaigh et al. [28]	3	Moderate
Ali et al. [29]	2	High
**Studies evaluating latent TB infection screening in recent TB contacts or prior to BCG vaccination**		
Higgins et al. [30]	2	High
Glynn et al. [31]	0	High
O’Donovan et al. [32]	0	High
O’Meara et al. [33]	6	Low
O’Sullivan et al. [34]	0	High
Bambury et al. [35]	0	High
Gaensbaeur et al. [36]	4	Moderate
Hennessy [37]	1	High
Tam et al. [38]	N/a	N/a
**Studies evaluating latent TB infection screening in vulnerable population groups**		
Millar et al. [39]	0	High
Doyle et al. [40]	5	Moderate
**Studies evaluating latent TB infection screening in health care workers**		
Ali et al. [29]	2	High
Smyth et al. [41]	1	High
Kelly et al. [42]	2	High
Power et al. [43]	0	High
Arya et al. [44]	N/a	N/a

*N/a* not applicable

These studies did report on the outcome of LTBI screening, including the prevalence of a positive screening test among the screened population. Therefore, they could not be assessed using the selected risk of bias tool

In the 11 studies evaluating LTBI screening in immunosuppressed patients, the risk of bias was high in six of 11 studies, moderate in four of 11 studies and low in one of 11 studies. In the two studies evaluating LTBI screening in people living with HIV, the risk of bias was high in one study and moderate in the other. In the eight studies evaluating LTBI screening in recent TB contacts, the risk was high in six of eight studies, moderate in one of eight studies and low in one of eight studies. In the two studies evaluating LTBI screening in asylum seekers, the risk of bias was moderate in one study and low in the other. All four studies which evaluated LTBI screening in health care workers had a high risk of bias. Aside from the risk of bias within studies, there is a risk of reporting bias across studies. Studies where any one or more of the offering, uptake and completion of screening or treatment for LTBI was poor may not have reported these outcomes.

### Latent TB infection screening and treatment outcomes in patients undergoing immunosuppression

Gnanasekaran et al. [17] was the only study that reported the proportion of the target population screened (95% of the target cohort) (Table 6). The median prevalence of a positive IGRA across all studies in this cohort was 7% (interquartile range (IQR) 7–8%). When considering only the studies where the risk of bias was moderate-low, the prevalence of a positive IGRA was 7% (IQR 5–7%). The median prevalence of a positive TST across all studies in this cohort was 17% (13–26%) and when considering only studies where the risk of bias was moderate-low, the median prevalence of a positive TST was 17% (IQR 15–18%).

**Table 6:** Results of studies evaluating LTBI in immunosuppressed patients

Study	Risk of bias score	Target sample size/proportion of target sample screened	Proportion with a positive screening test	Proportion offered prophylaxis	Proportion accepting prophylaxis	Proportion completing prophylaxis	Cost of screening/ treatment
Risk of bias moderate-low							
Gnanasekar-an et al. [17]	7	37/39 (95%)	IGRA + 2/37 (5%)	2/2	2/2	2/2	NR
Haroon et al. [23]	4	132	TST + 23/132 (17%)	23/23	23/23	14/23 (61%)	NR
O’Flynn [46]*	4	188	TST + 22/165 (13%)	33/33	33/33	NR	NR
			IGRA + 11/161 (7%)				
Martin et al. [25]	4	150	TST + 27/150 (18%)	NR	NR	NR	NR
			IGRA (T-SPOT) + 14/143 (10%)				
			IGRA (QFT) + 5/70 (7%)				
Kelly et al. [27]	4	101	IGRA + 5/71 (7%)	5/5	5/5	NR	NR
Risk of bias high							
O’Flynn et al. [18]	0	70	Unknown test 11/70 (16%)	NR	NR	NR	NR
Awan et al. [19]	0	25	TST + 3/25(12%)	3/3	3/3	3/3	NR
			IGRA + 2/25(8%)				
O’Flynn et al. [20]	2	109	IGRA + 9/109 (8%)	9/9	9/9	NR	NR
Hurley et al. [21]	1	101	TST + 9/101 (9%)	NR	NR	NR	NR
			IGRA + 8/101 (8%)				
Safwat et al. [22]	1	78	TST + 17/41 (41%)	NR	NR	NR	NR
			IGRA + 12/78 (15%)				
Jordan et al. [26]	0	63	TST + 21/63 (33%)	22/22	22/22	22/22	Cost of LTBI management 4 IGRA + = €1,652 18 TST + = €6,174
			IGRA + 4/63 (6%)				

*TST* tuberculin skin test; *IGRA* interferon gamma release assay; *QFT* quantiFERON; *NR* not reported

*TST performed first, IGRA then performed if TST negative

In all seven studies where the proportion of patients who were offered and accepted LTBI treatment was reported [17–20, 26, 27, 46], all patients were offered and accepted treatment. The median proportion of patients completing treatment was 100% (IQR 90–100%), with all patients in three studies [17, 19, 26] completing treatment, and 61% of patients in one study [23] completing treatment. Jordan et al. [26] reported the cost of treating four patients with LTBI diagnosed using an IGRA as €1652 and 21 patients diagnosed using a TST as €6174, although the methodology used to make these cost estimates is unclear.

### Latent TB infection screening and treatment outcomes in people living with HIV

Ni Cheallaigh et al. [28] reported the proportion of people living with HIV who had a positive test when screened using an IGRA as 18% when T-SPOT was used and 11% when QuantiFERON was used (sample sizes 256 and 247, respectively). When a TST was used among PLWHIV, the proportion of patients diagnosed with LTBI was 10% in the study by Ni Cheallaigh et al. [28] and 11% in the study by Ali et al. [29] (sample sizes 93 and 331 respectively). However, the risk of bias in the study by Ali et al. [29] was high. Ni Cheallaigh et al. [28] reported that all patients who were diagnosed with LTBI were offered treatment. No study reported on the proportion of patients completing LTBI treatment in this cohort.

### Latent TB infection screening and treatment outcomes in recent TB contacts or prior to BCG vaccination

Three studies reported on the proportion of the target sample screened as part of contact tracing with 97%, 83% and 79% respectively being screened (Table 7) [30, 31, 33, 35]. Seven studies reported on the proportion of patients diagnosed with LTBI in this cohort [30–36]. There were only two studies where the risk of bias was moderate-low and the screening test used was reported in the context of TB contact tracing. The study by O’Meara et al. described a TB outbreak in a primary school setting with 191 children screened using a TST [33]. Gaensbaeur et al. reported on two TB outbreaks in creches where 268 children were screened [36]. The prevalence of a positive TST in these studies was 9% and 7% respectively. One study reported on the proportion of recent TB contacts diagnosed with LTBI who were offered treatment, 61% [31]. Two studies reported on the proportion of TB case contacts accepting treatment as 31% and 67% [30, 35]. Two studies reported on the proportion of patients completing treatment as 33% and 77% [30, 35]. Two studies described the outcome of LTBI screening prior to BCG vaccination, one of which reported 13 cases of LTBI being offered treatment, 10 of whom accepted and completed treatment [38].

**Table 7:** Results of studies evaluating latent TB screening in recent TB contacts or prior to BCG vaccination

Study	Risk of bias score	Target sample size/proportion of target sample screened	Proportion screened positive	Proportion offered prophylaxis	Proportion accepting prophylaxis	Proportion completing prophylaxis	Cost of screening/treatment
Risk of bias moderate-low							
O’Meara et al. [33]	6	244/307 (79%)	TST 17/191 (9%)	NR	NR	NR	NR
Gaensbaeur et al. [36]	4	268	TST 20/268 (7%)	NR	NR	NR	NR
Risk of bias high							
Higgins et al. [30]	2	260/268 (97%)	TST 48/260 (19%)	NR	15/48 (31%)	NR	NR
Glynn et al. [31]	0	585/701 (83%)	Unknown test 76/585 (13%)	46/76 (71%)	NR	15/46 (33%)	NR
O’Donovan et al. [32]	0	71	Unknown test 26/71 (37%)	NR	NR	NR	NR
O’Sullivan et al. [34]	0	1200	TST > 60/1200 (> 5%)	NR	NR	NR	NR
Bambury et al. [35]	0	1082	Unknown 223/1082 (21%)	NR	150/223 (67%)	116/150 (77%)	NR
Hennessy [37]	1	NR	TST 13/1854 (< 1%)	NR	NR	NR	NR
Tam et al. [38]	N/a	NR	NR	13/13 (100%)	NR	10/13 (77%)	NR

*N/a* Not applicable, *NR* Not reported, *TST* tuberculin skin test

### Latent TB infection screening and treatment outcomes in asylum seekers

Millar et al. [39] reported on the proportion of the target sample screened in asylum seekers (28%) where screening was voluntary. Doyle et al. [40] reported that of 334 TSTs placed in a cohort of asylum seekers, only 236 were read. In this study, when screened using TST, 5/236 (2%) of those read were positive. Of these five patients, three were started on treatment. It is unclear if the remaining patients were not offered or declined LTBI treatment, and it is unclear how many completed treatment.

### Latent TB infection screening and treatment outcomes in health care workers

Five studies reported on LTBI screening in this cohort (Table 8) [29, 42–44, 46]. Two studies reported on the prevalence of LTBI in health care workers screened using a TST [29, 46]. In a cohort of new entrant health care workers, 32% had LTBI [29] while in health care workers with significant exposure to infectious TB, the prevalence was 56% [46]. Kelly et al. [42] reported that of new entrant health care workers from overseas were screened using a TST or an IGRA, 17% had a positive test result of which 85% were offered LTBI treatment [43]. Only 26% accepted treatment, all of whom completed treatment. Arya et al. [44] reported of 243 health care workers with a positive TST referred to a TB clinic, only 59% accepted LTBI treatment, but it is not reported how many were offered LTBI treatment. Of these, 62% completed treatment [44].

**Table 8:** Results of studies evaluating latent TB infection screening in health care workers

Study	Risk of bias score	Target sample size/proportion of target sample screened	Proportion screened positive	Proportion offered prophylaxis	Proportion accepting prophylaxis	Proportion completing prophylaxis	Cost of screening/treatment
Ali et al. [29]	2	2410	TST 765/2410 (32%)	NR	NR	NR	NR
Smyth et al. [41]	1	41	TST 23/42 (56%)	NR	NR	NR	NR
Kelly et al. [42]^a^	2	505	TST/IGRA 87/505 (17%)	74/87 (85%)	19/74 (26%)	19/19 (100%)	NR
Power et al. [43]^b^	0	54	TST 3/54 (6%)	NR	NR	NR	NR
Arya et al. [44]	N/a	243	243	NR	50/85 (59%)	31/50 (62%)	NR

*TST* tuberculin skin test, *IGRA* interferon-gamma release assay

*N/a* not applicable, *NR* not reported

^a^TST or IGRA

^b^Chest x-ray followed by TST (Mantoux) if abnormal

## Discussion

This research presents a comprehensive review of studies describing LTBI prevalence and screening and treatment outcomes in Ireland and highlights the significant knowledge gaps. The findings demonstrate that there are few studies that are reliably informative as to the prevalence of LTBI across all risk cohorts in Ireland. Studies were all performed on a local or regional level. When considering only the studies where the risk of bias was moderate-low, the prevalence of a positive IGRA among immunosuppressed patients was 7% (IQR 5–7%). There is no published research describing the prevalence of LTBI in people from countries with a high incidence of TB, people who are homeless, people in prisons and people who use intravenous drugs in Ireland, and for asylum seekers, there were only two studies describing the prevalence of LTBI, both of which had a moderate or high risk of bias. Regarding health care workers, only two studies, both performed in the same centre and both with a high risk of bias, were informative as to the prevalence of LTBI. Despite these cohorts having an increased risk of TB in other low-incidence countries [47–49], it is unclear if the incidence of TB in these cohorts in Ireland is high because TB cases are not described according to these characteristics in recent national surveillance reports. However, a 2015 report describing risk factors for TB cases notified in 2013 reported that approximately 20 to 25% of cases had “high endemicity residence”, approximately 30% had “high endemicity origin” and approximately 10% had “substance abuse” [50]. Additionally, significant TB outbreaks have been reported in the Irish prison system within the previous decade [51]. Studies assessing the prevalence of LTBI and risk of TB reactivation in people from countries with a high incidence of TB, people who use drugs and prison inmates should be a future research priority in Ireland.

Research describing the cascade of LTBI care in Ireland was limited. Among immunosuppressed patients, treatment acceptance and completion appeared to be generally high, although the number of patients with LTBI described in these studies was small. Among TB case contacts, provider recommendation of treatment was reported as 61% in one study [31], treatment acceptance was reported as 31% and 67% [30][30], and treatment completion was reported as 33% and 77% [31, 35]. Among health care workers, two studies reported that the acceptance of LTBI treatment was generally low. There was insufficient information in the literature to describe the cascades of care in other cohorts and provide insight into where it should be improved. There were no studies which described the cost-effectiveness of LTBI screening and treatment, which are important if LTBI is to be managed programmatically at scale. A 2015 report describing risk factors for TB cases notified in 2013 reported that approximately 10%, 5% and 5% of TB cases occurred in TB case contacts, people with immunosuppressive illnesses and people on immunosuppressive medications, respectively [50]. Studies evaluating the cascade of LTBI care in PLWHIV should be prioritised, and further studies evaluating the cascade of LTBI care in patients on immunosuppressive treatments and TB case contacts should be encouraged. These studies would have utility when defining the diagnostic algorithm most appropriate for each target group in Ireland, which is key for effective programmatic management [4].

The strengths of this review are its rigorous methodology and that it is the first comprehensive review of TB research in Ireland, which establishes with certainty that scope and degree of research are needed. A weakness of this systematic review was that the research question, while was intentionally broad, could have been more explicitly defined at inception using the PICO model. With regard to abstract publications, the authors were not contacted to search for any further results. However, most abstracts included in this review described single-centre studies with a small sample size obtained using convenience sampling, limiting their utility when assessing the prevalence of LTBI. A limitation of this research was that there were few studies which were reliably informative as to the prevalence of LTBI because the risk of sampling bias was high across almost all studies. Therefore, the limited prevalence estimates reported in this review should be interpreted with caution.

Intensified research and innovation is a strategic pillar of the WHO End TB strategy, which should be adapted at a country level with global collaboration [2]. Studies meeting the identified research needs must be performed. The WHO describes the components of an enabling environment for high-quality research, which has relevance for LTBI research in Ireland [52, 53]. These components include having a national TB research network. This could enhance collaboration between researchers, health care providers and patients and coordinate local and national TB research activities to align with national TB programme priorities [52]. The WHO recommends the formation of a country-specific TB research agenda and strategic plan to guide country-specific actions [52]. Other low TB incidence countries have advanced national LTBI research in cohorts they have identified as at risk, such as in Canada and England, where LTBI research priorities have been outlined in TB elimination strategies [14, 54]. In Canada, the Public Health Agency have funded studies in Inuit people [55–57] and in the UK, Public Health England [8, 11], the National Institute of Health Research [8, 10, 11, 58]and the Medical Research Council [9, 10] have funded LTBI research in people from countries with a high incidence of TB. TB research networks must not only contribute to local and national TB elimination efforts but also global TB elimination efforts through international collaboration [2]. Other European countries such as the Netherlands are prime examples of how countries with a low incidence of TB can be global leaders in transnational collaboration for TB research by funding and developing in their institutions TB researchers and research programmes that are guided by a national TB research agenda and the WHO Global TB Research Agenda [59].

The WHO advises that an enabling environment for TB research should have sufficient local researchers with the necessary profiles in TB research and incentives to retain them in employment and that there should be specialized training on TB for new researchers [52]. Although there are many researchers involved in other aspects of TB in Ireland, such as host–pathogen response, drug development and TB diagnostics [60, 61], such is the scale of the identified LTBI epidemiological and cascade of care research needs that to meet them, dedicated TB research positions should be created within research institutions and form part of a TB-network. A high-quality research network with a well-defined research plan and strategy and the opportunity for international collaboration could attract new researchers to this field in Ireland and contribute to achieving TB elimination. The research needs identified in this systematic review would be best met by inclusion in a TB research agenda and strategic plan and delivered through a TB-network that develops local, national and international TB research.

## Conclusion

This systematic review has described what published research there is on the epidemiology and cascade of LTBI care in Ireland and identified knowledge gaps. A strategy for addressing these knowledge gaps has been proposed.

## Appendix 1. PRISMA checklist

**Table:** 

Section/topic	No	Checklist item	Reported on page no
**TITLE**			
Title	1	Identify the report as a systematic review, meta-analysis, or both	1
**ABSTRACT**			
Structured summary	2	Provide a structured summary including, as applicable: background; objectives; data sources; study eligibility criteria, participants, and interventions; study appraisal and synthesis methods; results; limitations; conclusions and implications of key findings; systematic review registration number	2
**Introduction**			
Rationale	3	Describe the rationale for the review in the context of what is already known	3–4
Objectives	4	Provide an explicit statement of questions being addressed with reference to participants, interventions, comparisons, outcomes, and study design (PICOS)	4
**Methods**			
Protocol and registration	5	Indicate if a review protocol exists, if and where it can be accessed (e.g., Web address), and, if available, provide registration information including registration number	5
Eligibility criteria	6	Specify study characteristics (e.g., PICOS, length of follow-up) and report characteristics (e.g., years considered, language, publication status) used as criteria for eligibility, giving rationale	5
Information sources	7	Describe all information sources (e.g., databases with dates of coverage, contact with study authors to identify additional studies) in the search and date last searched	5
Search	8	Present full electronic search strategy for at least one database, including any limits used, such that it could be repeated	Appendix 2
Study selection	9	State the process for selecting studies (i.e., screening, eligibility, included in systematic review, and, if applicable, included in the meta-analysis)	Page 5 and Appendix 2
Data collection process	10	Describe method of data extraction from reports (e.g., piloted forms, independently, in duplicate) and any processes for obtaining and confirming data from investigators	Page 5 and Appendix 2
Data items	11	List and define all variables for which data were sought (e.g., PICOS, funding sources) and any assumptions and simplifications made	5
Risk of bias in individual studies	12	Describe methods used for assessing risk of bias of individual studies (including specification of whether this was done at the study or outcome level), and how this information is to be used in any data synthesis	Page 5, Appendix 2 and 3
Summary measures	13	State the principal summary measures (e.g., risk ratio, difference in means)	5
Synthesis of results	14	Describe the methods of handling data and combining results of studies, if done, including measures of consistency	5
Risk of bias across studies	15	Specify any assessment of risk of bias that may affect the cumulative evidence (e.g., publication bias, selective reporting within studies)	Appendix 3
Additional analyses	16	Describe methods of additional analyses (e.g., sensitivity or subgroup analyses, meta-regression), if done, indicating which were pre-specified	No additional analyses
**Results**			
Study selection	17	Give numbers of studies screened, assessed for eligibility, and included in the review, with reasons for exclusions at each stage, ideally with a flow diagram	Figure 1
Study characteristics	18	For each study, present characteristics for which data were extracted (e.g., study size, PICOS, follow-up period) and provide the citations	Page 6 and Table 3
Risk of bias within studies	19	Present data on risk of bias of each study and, if available, any outcome level assessment (see item 12)	Page 8 and Table 4
Results of individual studies	20	For all outcomes considered (benefits or harms), present, for each study: (a) simple summary data for each intervention group (b) effect estimates and confidence intervals, ideally with a forest plot	Pages 10–14
Synthesis of results	21	Present results of each meta-analysis done, including confidence intervals and measures of consistency	Pages 10–14
Risk of bias across studies	22	Present results of any assessment of risk of bias across studies (see Item 15)	Appendix 3
Additional analysis	23	Give results of additional analyses, if done (e.g., sensitivity or subgroup analyses, meta-regression [see Item 16])	No additional analyses
**Discussion**			
Summary of evidence	24	Summarize the main findings including the strength of evidence for each main outcome; consider their relevance to key groups (e.g., healthcare providers, users, and policy makers)	Pages 14–15
Limitations	25	Discuss limitations at study and outcome level (e.g., risk of bias), and at review-level (e.g., incomplete retrieval of identified research, reporting bias)	Pages 14–15
Conclusions	26	Provide a general interpretation of the results in the context of other evidence, and implications for future research	Page 15
**Funding**			
Funding	27	Describe sources of funding for the systematic review and other support (e.g., supply of data); role of funders for the systematic review	Page 16

## Appendix 2. Protocol for a systematic review of studies evaluating latent TB screening in the Republic of Ireland

## Introduction

Latent tuberculosis infection (LTBI) is a state of a persistent immune response to stimulation by *Mycobacterium tuberculosis* antigens with no evidence of clinically manifest active tuberculosis (TB) [299]. It is estimated that 24.8% of the world’s population has LTBI [465]. In high-income low-incidence TB countries, most TB disease occurs due to the reactivation of latent TB and not ongoing disease transmission [586]. The World Health Organization’s (WHO) End TB Strategy states that the identification and management of LTBI in groups of people at high risk of reactivation is an essential part of TB elimination in low-incidence countries [18]. The End TB Strategy also suggests that epidemiological research should be conducted to determine the burden of LTBI in various geographical settings and risk groups and as a basis for nationally and locally tailored interventions, including integrated community-based approaches [18]. The *Health Protection Surveillance Centre Guidelines for the Prevention and Control of TB 2010* guidelines outline which groups should be prioritized for LTBI screening in Ireland and offer guidance as to the diagnostic approach for certain target groups (Table 9) [151]. However, these guidelines do not discuss strategies for service delivery and programmatic monitoring and evaluation. In this systematic review, we aim to determine what evidence exists to describe the epidemiology of LTBI in the Republic of Ireland. Knowledge of regional LTBI epidemiology is crucial to improve the programmatic management of LTBI.

**Table 9:** Groups to be prioritized for LTBI screening in the Republic of Ireland

	All age groups		All those aged ≤ 35 years of age or ≤ 55 years of age if supervised directly observed therapy is available
1	Recent converters	5	Persons from countries with high TB endemicity
2	HIV-positive individuals	6	People who are homeless
3	Persons receiving immunosuppressive therapy	7	People who use intravenous drugs
4	Persons with evidence of old healed TB lesions on chest X-ray	8	Health care workers

### Aim

We aim to describe the epidemiology of LTBI in the Republic of Ireland including its prevalence, screening outcomes, and treatment outcomes.

### Objectives

To determine:

1. What the prevalence of LTBI is in the Republic of Ireland
2. What proportion of patients are being offered treatment
3. What proportion of patients are accepting treatment
4. What proportion of patients are completing treatment
5. What the cost of LTBI screening is in the Republic of Ireland

### Methods

A systematic literature review will be performed and reported in adherence with the Preferred Reporting Items for Systematic Reviews and Meta-Analysis (PRISMA) statement [463].

#### Included studies

We will include all studies describing patients who were screened for LTBI in the Republic of Ireland. Studies must be published in English. Conference abstracts will be included for review. All studies published from 01 Jan. 2000 to 31 Dec. 2020 will be included. Studies must use any one or a combination of chest X-ray (CXR), tuberculin skin tests (TST) or interferon-gamma release assay (IGRA) to screen for LTBI. Clinical audits randomized controlled trials, diagnostic accuracy studies, retrospective cohort reviews, and prospective cohort reviews will be included. We plan to exclude any studies where it was not possible to ascertain data on only patients screened in the Republic of Ireland.

#### Outcomes

The outcomes chosen are screening test used, the proportion of people screened out of target population, the prevalence of a positive screening test in the target population, proportion of those diagnosed with LTBI who are offered treatment, the proportion of those diagnosed with LTBI who started treatment for LTBI, the proportion of those diagnosed with LTBI who completed treatment and the cost of performing screening and/or treatment of LTBI cases identified.

#### Search methods

We will search MEDLINE (via OVID), Embase, Web of Science and Google Scholar. The references of the included studies will also be searched. All available published conference abstracts will be searched from the Irish Thoracic Society (published online in the *Irish Journal of Medical Science*), Irish Society of Rheumatology (published online in the *Irish Journal of Medical Science*), Irish Society of Gastroenterology (published online in the *Irish Journal of Medical Science*), Royal College of Physicians Ireland Faculty of Public Health and Faculty of Occupational Medicine (published online in the *Irish Journal of Medical Science*), the Infectious Diseases Society of Ireland (published online at www.idsi.ie) and the Irish Nephrology Society (www.nephrology.ie). A full list of conference abstracts is to be searched.

#### Search strategy

The search strategy, including a full list of the conference abstracts to be searched, is shown below. Both free-text terms and MeSH terms will be used in EMBASE, Medline and Web of Science. O’Connell J. designed the search strategy. The search will be performed independently by O’Connell J. and Gibbons C. All records returned will be screened by O’Connell J. and Gibbons C. independently. All records which are deemed to meet the study inclusion criteria will then have their full articles reviewed. All articles included for full-text review will have their references searched for other studies that meet inclusion criteria.

Embase search strategy.

**Table:** 

1	‘tuberculosis’/exp OR ‘tuberculosis’
2	‘mycobacterium tuberculosis’/exp OR ‘mycobacterium tuberculosis’
3	‘latent tuberculosis’/exp OR ‘latent tuberculosis’
4	#1 OR #2 OR #3
5	‘ireland’/exp OR ‘ireland’
6	‘screening’/exp OR ‘screening’
7	‘microorganism detection’/exp OR ‘microorganism detection’
8	‘assessment of humans’/exp OR ‘assessment of humans’
9	#6 OR #7 OR #8
10	(‘interferon’/exp OR interferon) AND gamma AND (‘release’/exp OR release) AND (‘assay’/exp OR assay)
11	‘tuberculin test’/exp OR ‘tuberculin test’
12	#10 OR #11
13	#4 AND #5 AND #9 AND #12

### MEDLINE (OVID) search strategy

1. exp Mycobacterium tuberculosis/
2. exp Tuberculosis/
3. exp Latent Tuberculosis/
4. 1 or 2 or 3
5. (“tuberculosis” or “mycobacterium tuberculosis” or “latent tuberculosis”).mp. [mp = title, abstract, original title, name of substance word, subject heading word, floating sub-heading word, keyword heading word, organism supplementary concept word, protocol supplementary concept word, rare disease supplementary concept word, unique identifier, synonyms].
6. exp Ireland/
7. (ireland or Irish).mp. [mp = title, abstract, original title, name of substance word, subject heading word, floating sub-heading word, keyword heading word, organism supplementary concept word, protocol supplementary concept word, rare disease supplementary concept word, unique identifier, synonyms].
8. (screen* or test* or assess*).mp. [mp = title, abstract, original title, name of substance word, subject heading word, floating sub-heading word, keyword heading word, organism supplementary concept word, protocol supplementary concept word, rare disease supplementary concept word, unique identifier, synonyms].
9. exp Mass Screening/
10. “interferon-gamma release assay”.mp. [mp = title, abstract, original title, name of substance word, subject heading word, floating sub-heading word, keyword heading word, organism supplementary concept word, protocol supplementary concept word, rare disease supplementary concept word, unique identifier, synonyms].
11. exp Interferon-gamma Release Tests/
12. exp Tuberculin Test/
13. (“tuberculin” or “mantoux”).mp. [mp = title, abstract, original title, name of substance word, subject heading word, floating sub-heading word, keyword heading word, organism supplementary concept word, protocol supplementary concept word, rare disease supplementary concept word, unique identifier, synonyms].
14. 4 or 5.
15. 6 or 7.
16. 8 or 9.
17. 10 or 11 or 12 or 13.
18. 14 and 15 and 16 and 17.

### Web of Science search strategy

1. TS = (Tuberculosis OR Latent Tuberculosis OR Mycobacterium Tuberculosis).
2. TS = (Tuberculosis OR Latent NEAR/1 Tuberculosis OR Mycobacterium NEAR/1 Tuberculosis).
3. TS = (Screen* OR Assess* OR Detect*).
4. ALL = (‘Interferon Gamma Release Assay’ OR Mantoux OR Tuberculin).
5. #2 OR #1 [ ALL TB]
6. #4 OR #3 [ALL SCREEN OR TEST]
7. #6 AND #5 [TB AND SCREEN].
8. TS = (Ireland OR Irish).
9. #8 AND #7 [TB IRELAND].
10. ALL = (veterinary OR livestock OR agricultur* OR cattle OR sheep OR pigs OR chickens OR avian).
11. #9 NOT #10 [REMOVE LIVESTOCK]

#### Google Scholar search strategy

[Tuberculosis OR TB OR Mycobacterium Tuberculosis] AND [Ireland OR Irish] AND [Screen* OR Detect* OR Assess* OR Test*] AND [Tuberculin OR Mantoux OR Interferon Gamma Release Assay].

### Conference Abstract Search Strategy

The conference abstract publications identified for searching are shown in Table 10.

**Table 10:** Conference Abstract Publications Identified for Searching

Publication
**Royal College of Physicians of Ireland Faculty of Public Health Summer and Winter Scientific Meetings**
1.Irish Journal of Medical Science, December 2016, Issue 12 Supplement, Pages 527–561 Proceedings of the Faculty of Public Health Medicine, Summer & Winter Scientific Meetings 2015 2. Irish Journal of Medical Science, August 2012, Issue 5 Supplement, Pages 121–127 Faculty of Public Health Medicine, Royal College of Physicians of Ireland—Summer Scientific Meeting, 23rd—24th May, 2012, Dublin 3. Irish Journal of Medical Science, August 2012, Issue 4 Supplement, Pages 109–119 Faculty of Public Health Medicine, Royal College of Physicians of Ireland—Winter Scientific Meeting Abstracts 14th December, 2011, Dublin 4. Irish Journal of Medical Science July 2011, Issue 7 Supplement, Pages 221–232 Faculty of Public health Medicine Summer Scientific meeting, 25th & 26th May 2011, RCPI, Dublin 5. Irish Journal of Medical Science June 2011, Issue 6 Supplement, Pages 213–220 Faculty of Public Health Medicine, Winter Scientific Meeting, RCPI Dublin, 8th Dec 2010 6. Irish Journal of Medical Science October 2010, Issue 11 Supplement, Pages 413–446 The Summer 2008, Winter 2008, Summer 2009 & Winter 2009 Scientific Meetings of the Faculty of Public Health Medicine of the Royal College of Physicians of Ireland 7. Irish Journal of Medical Science August 2010, Issue 8 Supplement, Pages 303–311 Summer Scientific Meeting of the Faculty of Public Health Medicine of the Royal College of Physicians of Ireland, Dublin, 24 & 25th May 2010 8. Irish Journal of Medical Science, Volume 169, Issue 4 Supplement, April 2000, Faculty of Public Health Medicine Summer Scientific Meeting 1999
**Health Service Executive**
Irish Journal of Medical Science, October 2016, Issue 8 Supplement, Pages 421–437 2nd Annual Multidisciplinary Galway University Hospitals Research Symposium, 2016
**Infectious Diseases Society of Ireland**
Annual Scientific Meetings Abstracts 2011- 2019, www.idsociety.ie
**Irish Nephrology Society**
Annual Scientific Meeting 2018, https://nephrology.ie/ins-annual-meeting/
**Royal Academy of Medicine in Ireland**
1. Irish Journal Medical Science 188, 31–127 (2019). https://doi.org/10.1007/s11845-019-02053-0 Proceedings of the Intern Section of the Royal Academy of Medicine in Ireland (RAMI) Venue: Mater, Dublin 7 on Saturday 2^nd^ February 2019 2. Irish Journal of Medical Science, March 2018, Issue 3 Supplement, Pages 17–113 Proceedings of the RAMI Intern Section Meeting, Saturday 27 January 2018 3. Irish Journal of Medical Science, June 2017, Issue 6 Supplement, Pages 171–280 Proceedings of the RAMI Intern Section Meeting, 14 January 2017 4. Irish Journal of Medical Science, June 2016, Issue 5 Supplement, Pages 187–299 Proceedings of the RAMI Section of Interns Study Day Saturday 30th January 2016 5. Irish Journal of Medical Science, July 2015, Volume 184, Supplement 7, pp 249–344 | RAMI Intern Section Meeting held on 31st January 2015 6. Irish Journal of Medical Science, July 2014, Issue 4 Supplement, Pages 119–199 Proceedings of the RAMI Intern Section Meeting, 18th January 2014 7. Irish Journal of Medical Science June 2013, Issue 5 Supplement, Pages 143–178 Proceedings of the RAMI Section of Interns Study Day, 26th January 2013, Royal College of Physicians of Ireland 8. Irish Journal of Medical Science July 2012, Issue 3 Supplement, Pages 83–107 Proceedings of the RAMI Section of Interns Study Day, 21st April 2012 9. Irish Journal of Medical Science Volume 170, Issue 3 Supplement, October–December 2001, Royal Academy of Medicine in Ireland Jacqueline Horgan Epidemiology Prize November 2001 10. Irish Journal of Medical Science, Volume 169, Issue 4 Supplement, April 2000, Royal Academy of Medicine in Ireland Jacqueline Horgan Epidemiology Prize 1999
**Irish Society of Gastroenterology**
1. Irish Journal of Medical Science February 2015, Issue 3 Supplement, Pages 67–102 Irish Society of Gastroenterology, Summer Meeting, 12th and 13th June 2014 2. Irish Journal of Medical Science, Volume 184, Issue 6 Supplement, June 2015, Irish Society of Gastroenterology – Winter Meeting 2014 3. Irish Journal of Medical Science, February 2015, Issue 2 Supplement, Pages 19–65 Irish Society of Gastroentrology, Winter Meeting, 22nd and 23rd November 2013
Irish Society of Rheumatology
1. Irish Journal of Medical Science, April 2014, Issue 3 Supplement, Pages 87–118 Irish Society for Rheumatology, Autumn Meeting 2013, 19th & 20th September 2013 2. Irish Journal of Medical Science, June 2012, Issue 2 Supplement, Pages 49–81 Irish Society for Rheumatology, Autumn Meeting 2011, 29th & 30th September 2011 3. Irish Journal of Medical Science, June 2011, Issue 5 Supplement, Articles 169–169 Irish Society of Rheumatology Autumn Scientific Meeting 2010 4. Irish Journal of Medical Science, November 2010, Issue 14 Supplement, Pages 539–574 Irish Society for Rheumatology & Irish Rheumatology Health Professionals Society – Autumn Scientific Meeting 2009 5. Irish Journal of Medical Science March 2008, Issue 3 Supplement, Pages 71–108 Irish Society for Rheumatology (ISR) and Irish Rheumatology Health Professionals Society (IRHPS) combined AGM 2007 6. Irish Journal of Medical Science, Volume 169, Issue 4 Supplement, April 2000, Irish Society for Rheumatology Meeting October 1999
**Irish Thoracic Society**
1. Irish Journal of Medical Science 188, 255–320 (2019). Irish Thoracic Society Annual Scientific Meeting 2019 2. Irish Journal of Medical Science, August 2018, Issue 8 Supplement, Pages 237–303 Irish Thoracic Society Annual Scientific Meeting, 23rd–24th November 2018 3. Irish Journal of Medical Science, October 2017, Issue 10 Supplement, Pages 387–445 Irish Thoracic Society Annual Scientific Meeting 2017, 10th–11th November 2017 4. Irish Journal of Medical Science, November 2016, Issue 9 Supplement, Pages 439–508, ITS Annual Scientific Meeting 2016 5. Irish Journal of Medical Science, Irish Thoracic Society Annual Scientific Meeting 2017, 10th–11th November 2017 6. Irish Thoracic Society Annual Scientific Meeting 2015, 13th–14th November 2015, Issue 11 Supplement, Pages 475–547 7. Irish Journal of Medical Science, Volume 183, Issue 11 Supplement, November 2014, Irish Thoracic Society Annual Scientific Meeting 2014 8. Irish Journal of Medical Science, November 2013, Issue 10 Supplement, Pages 427–486 Irish Thoracic Society Annual Scientific Meeting 2013, 15th – 16th November 9. Irish Journal of Medical Science, November 2012, Issue 10 Supplement, Pages 369–437 Irish Thoracic Society Annual Scientific Meeting 2012, 23rd – 24th November, Limerick, Ireland 10. Irish Journal of Medical Science, November 2011, Issue 12 Supplement, Articles 411–411 Irish Thoracic Society Annual Scientific Meeting 2011, 11th -12th November, 11. Irish Journal of Medical Science, November 2009, Issue 11 Supplement, Articles 423–423 Irish Thoracic Society Annual Scientific Meeting 2009 12. Irish Journal of Medical Science, November 2008, Issue 13 Supplement, Pages 425–482 Irish Thoracic Society Annual Scientific Meeting, 2008 13. Irish Journal of Medical Science, November 2007, Issue 10 Supplement, Pages 385–426 Irish Thoracic Society Annual Scientific Meeting 2007 14. Irish Journal of Medical Science, Volume 170, Issue 3 Supplement, October–December 2001 Irish Thoracic Society Annual Scientific Meeting 2000 15. Irish Journal of Medical Science Volume 169, Issue 4 Supplement, April 2000, Irish Thoracic Society Annual Scientific Meeting 1999 16. Irish Journal of Medical Science April 2000, 169:24 Irish Thoracic Society Annual Scientific Meeting 13th & 14th November 1998

**Table 11:** Data points collected for systematic review literature

Datapoint collected	Description
Screening test used	Test used to determine if further assessment was necessary to confirm or deny a diagnosis of latent TB Included IGRA, TST, CXR
Indication for screening	The reason why LTBI screening was performed
The proportion of people screened out of target sample population	The proportion of people screened out of the total group of people targeted as defined by the authors
The proportion of patients screened with a positive test	The proportion of all patients screened who have a positive chest X-ray, TST, or IGRA
The proportion of patients with a positive test offered LTBI treatment	
The proportion of patients offered LTBI treatment who accepted treatment	
The proportion of patients on treatment for LTBI who completed treatment	
The cost of screening for LTBI and or treatment	

#### Risk of bias assessment

We will perform a risk of bias assessment using a tool designed for assessing the risk of bias in TB prevalence studies (Appendix 3), which was based on guidance from Cochrane and an existing risk of bias tool for prevalence studies [465]. The risk of bias assessment will be performed by O’Connell J. and Gibbons C. Any disagreements in the risk of bias assessment of studies included will be resolved by mutual agreement.

#### Data extraction

Data extraction will be performed by two reviewers, O’Connell J. and Li B. Both authors will extract the data independently into a data collection tool. Any disagreement in data extracted will be resolved by discussion and mutual agreement. Data points for extraction for shown in Table 11. 

References

1. Rosales-Klintz S, Bruchfeld J, Haas W, Heldal E, Houben RM, van Kessel F, et al. Guidance for programmatic management of latent tuberculosis infection in the European Union/European Economic Area. Eur Respiratory Soc; 2019.

2. Cohen A, Mathiasen VD, Schӧn T, Wejse C. The global prevalence of latent tuberculosis: a systematic review and meta-analysis. European Respiratory Journal. Eur Respiratory Soc; 2019;54(3).

3. France AM, Grant J, Kammerer JS, Navin TR. A field-validated approach using surveillance and genotyping data to estimate tuberculosis attributable to recent transmission in the United States. American journal of epidemiology. Oxford University Press; 2015;182(9):799–807.

4. Uplekar M, Weil D, Lonnroth K, Jaramillo E, Lienhardt C, Dias HM, et al. WHO’s new end TB strategy. The Lancet. Elsevier; 2015;385(9979):1799–801.

5. National Tuberculosis Advisory Committee, Guidelines on the Prevention and Control of Tuberculosis in Ireland 2010. Health Protection Surveillance Centre (HPSC); 2010.

6. Moher D, Liberati A, Tetzlaff J, Altman DG. Preferred Reporting Items for Systematic Reviews and Meta-Analyses: The PRISMA Statement. Journal of Clinical Epidemiology. 2009;62(10):1006–12.

## Appendix 3. Risk of bias assessment tool

For the prevalence of a positive screening test, we assessed the risk of bias using a tool designed for TB prevalence studies that was derived from on an existing tool for prevalence studies (Table 12) [354]. The tool assesses the risk of bias across four domains, and each domain is scored on a scale of 0–2. A maximum score of 8 can be given for studies which score a low risk of bias across all domains. A minimum score of 0 can be given for studies which score a high risk of bias across all domains. The risk of bias assessment was performed independently by O’Connell J. and Gibbons C. Any disagreements in the risk of bias assessment of studies included were resolved by mutual agreement. The outcome of the risk of bias assessment is shown in full in Table 13.

**Table 12:** Risk of bias assessment tool

Domain and Score	Criteria
Quality of sampling method	
0	A convenience sample of the target population was used
1	A random sample of the target population was used
2	A national survey or multisite random sample of the target population was used
Quality of selection method	
0	There were no exclusion criteria stated or a risk factor for LTBI was an exclusion criterion
1	Exclusion criteria were stated and a risk factor for LTBI was not an exclusion criteria
2	The means of identification of TB was stated
Response rate	
0	Not reported
1	The proportion of the sample screened is reported and is under 65%
2	The proportion of the sample screened is reported and is 65% or above
Quality of prevalence assessment	
0	TST cut-off at 10 mm was not present/Indeterminate IGRA results were not stated
1	TST cut-off at 10 mm was present/ Indeterminate IGRA results were stated
2	TST cut-off at 5 or 15 mm was present as well/ Indeterminate IGRA results constituted < 10%

**Table 13:** Risk of bias assessment outcome.

Study	Quality of sampling method	Quality of selection method	Response rate	Quality of prevalence assessment	Total risk of bias score	Risk of bias
**Studies evaluating latent TB infection screening in patients undergoing immunosuppression**						
Gnanasekaran et al. [357]	1	2	2	2	7	Low
O’Flynn et al. [358]	0	0	0	0	0	High
Awan et al. [359]	0	0	0	0	0	High
O’Flynn et al. [360]	0	2	0	0	2	High
Hurley et al. [361]	0	0	0	1	1	High
Safwat et al. [362]	0	0	0	1	1	High
Haroon et al. 2012 [363]	0	2	2	0	4	Moderate
O’Flynn [402]	0	2	0	2	4	Moderate
Martin et al. [365]	0	2	0	2	4	Moderate
Jordan et al. [366]	0	0	0	0	0	High
Kelly et al. [367]	0	2	2	0	4	Moderate
**Studies evaluating latent TB infection screening in people living with HIV**						
Ni Cheallaigh et al. [368]	0	1	0	2	3	Moderate
Ali et al. [369]	0	2	0	0	2	High
**Studies evaluating latent TB infection screening in recent TB contacts or prior to BCG vaccination**						
Higgins et al. [370]	0	0	2	0	2	High
Glynn et al. [371]	0	0	0	0	0	High
O’Donovan et al. [372]	0	0	0	0	0	High
O’Meara et al. 2005 [373]	0	2	2	2	6	Low
O’Sullivan et al. 2000 [374]	0	1	0	0	0	High
Bambury et al. [375]	0	0	0	0	0	High
Gaensbaeur et al. [376]	0	2	2	0	4	Moderate
Hennessy [377]	0	1	0	0	1	High
Tam et al. [378]	N/a	N/a	N/a	N/a	N/a	N/a
**Studies evaluating latent TB infection screening in vulnerable population groups**						
Millar et al. [379]	0	0	0	0	0	High
Doyle et al. [380]	0	2	2	1	5	Moderate
**Studies evaluating latent TB infection screening in healthcare workers**						
Ali et al. [369]	0	2	0	0	2	High
Smyth et al. [403]	0	0	0	1	1	High
Kelly et al. [381]	0	2	0	0	2	High
Power et al. [382]	0	0	0	0	0	High
Arya et al. [404]	N/a	N/a	N/a	N/a	N/a	N/a

These studies did report on the outcome of LTBI screening including the prevalence of a positive screening test among the screened population. Therefore, they could not be assessed using the selected risk of bias tool

*N/a* not applicable

The risk of bias relating to the other outcomes in the cascade of care was not formally assessed with a risk of bias tool because most of the items in risk of bias tools for non-randomized studies, such as the ROBINS-I tool [355] or the Newcastle Ottawa Scale [356], are not applicable to the primarily descriptive non-interventional studies that describe the cascade of TB care, and this limitation has always been encountered in other systematic reviews of TB cascades of care [291].
